# New means to assess neonatal inflammatory brain injury

**DOI:** 10.1186/s12974-015-0397-2

**Published:** 2015-09-25

**Authors:** Chen Jin, Irene Londono, Carina Mallard, Gregory A. Lodygensky

**Affiliations:** Department of Pediatrics, Sainte-Justine Hospital and Research Center, Université de Montréal, 3175 Chemin de la Côte-Sainte-Catherine, Montréal, Québec H3T 1C5 Canada; Perinatal Center, Institute of Neuroscience and Physiology, Sahlgrenska Academy, University of Gothenburg, 405 30 Gothenburg, Sweden; Montreal Heart Institute, 5000 Rue Bélanger, Montréal, Québec Canada; Department of Neuroscience and Pharmacology, Université de Montréal, Montréal, Québec Canada

**Keywords:** Brain injury, White matter injury, MRI, ADC, Biomarker, Inflammation

## Abstract

Preterm infants are especially vulnerable to infection-induced white matter injury, associated with cerebral palsy, cognitive and psychomotor impairment, and other adverse neurological outcomes. The etiology of such lesions is complex and multifactorial. Furthermore, timing and length of exposure to infection also influence neurodevelopmental outcomes. Different mechanisms have been posited to mediate the observed brain injury including microglial activation followed by subsequent release of pro-inflammatory species, glutamate-induced excitotoxicity, and vulnerability of developing oligodendrocytes to cerebral insults. The prevalence of such neurological impairments requires an urgent need for early detection and effective neuroprotective strategies. Accordingly, noninvasive methods of monitoring disease progression and therapy effectiveness are essential. While diagnostic tools using biomarkers from bodily fluids may provide useful information regarding potential risks of developing neurological diseases, the use of magnetic resonance imaging/spectroscopy has emerged as a promising candidate for such purpose. Various pharmacological agents have demonstrated protective effects in the immature brain in animal models; however, few studies have progressed to clinical trials with promising results.

## Introduction

In 1867, Virchow first described the pathological changes in the neonatal brain, characterized by softening of the periventricular white matter, and employed the term “congenital encephalomyelitis” where he emphasized the inflammatory nature of the disease [1]. In reference to the same pathology, a new term was later introduced, namely periventricular leukomalacia (PVL), by Banker and Larroche in 1962 [2]. Volpe later noted that such neurological anomaly is not only marked by the presence of periventricular white matter injury but is also observed along with neuronal/axonal damage in the brain including, but not limited to, areas of the cerebral cortex, cerebral white matter, basal ganglia, and thalamus, more appropriately introducing the term “encephalopathy of prematurity” [3, 4].

There is evidence for an association between antenatal infection with subsequent neurological injuries including perinatal white matter injury, cerebral palsy, blindness, deafness, and motor and cognitive deficits [5–10]. However, evidence has also emerged suggesting that antenatal exposure to infection may not be directly associated with increased risk of adverse outcomes [11–14] and may, in fact, reduce the risk of death and neurodevelopmental deficits in the long run [15, 16]. To study such complex effects of the interplay of timing and length of exposure to inflammation and its consequences on neurodevelopment, it is important to determine biomarkers that are specific, robust, and detectable at an early stage. Animal models have been used to study the associated risk factors for adverse outcomes, both antenatal [17–23] and postnatal [24, 25] with only a few available imaging studies.

### Vulnerability of preterm infants to infection

In humans, maturation of the adaptive immune system occurs after birth, thus making the innate immune system largely responsible for fighting off infections in the first weeks of life [26, 27]. However, as the innate immune system develops around 24 weeks of gestation and continues until term [28], premature infants may have a reduced ability to respond to pathogens [26, 27, 29–31], characterized by reduced expression of surface innate immune receptors, and/or immature intracellular downstream responses [32, 33]. Conflicting evidence has been presented that the pro-inflammatory response in the neonates may not necessarily be reduced, as observed with similar or even higher levels of pro-inflammatory cytokines detected in preterm neonates compared to term neonates [34] or adults [35], but rather, a reduced anti-inflammatory response [36–39] or dysregulation of the immune system may play a major role in brain injury [40]. The assessment of detailed inflammatory mechanisms is essential in identifying robust markers that can be used to detect brain injury.

### Systemic inflammatory response possibly induces white matter injury

Pro-inflammatory cytokines are upregulated in the brain following peripheral lipopolysaccharide (LPS) administration in animal studies; however, mechanisms of how systemic inflammation relay signals to the brain that leads to increased central nervous system (CNS) inflammation and injury continues to be debated [41]. One proposed mechanism is through the direct transport of inflammatory agents or inflammatory cells across the blood–brain barrier (BBB). Saturable transport systems for pro-inflammatory cytokines in the BBB in mature murine models have also been detected [42, 43], suggesting that systemic pro-inflammatory cytokines may cross the BBB to elicit brain inflammation. In 1993, Leviton proposed that infection-induced upregulation of tumor necrosis factor alpha (TNF-α) can produce brain injury [44]. The hypothesis was supported by various studies later demonstrating that placental production of pro-inflammatory cytokines such as TNF-α [42], interleukin (IL)-1α, IL-1β [45], and IL-6 [43] can cross the BBB. On the other hand, systemic LPS given to P5 mice induces marked microglia proliferation but with no evidence of contribution of peripheral myeloid monocytes or granulocytes to the brain inflammation [46]. The proposition that bacterial products, such as LPS, cross the BBB to induce CNS inflammation directly is less likely as studies show that systemic LPS was mostly found to be concentrated in the brain endothelial cells and not inside the brain [47, 48].

Studies have suggested that the BBB may not develop until birth, as it was assumed that such development was associated with the appearance of astrocytes, which do not appear until after birth [49, 50]. A group of scientists recently suggested that BBB development may start in earlier embryonic stages of development, based on the evidence that pericytes, rather than astrocytes, may be responsible for such process [51]. Difference in diffusion characteristics of BBB between developing and the mature brain has been noted, possibly due to the specific fetal environment [52]. Such differences in structure/morphological features may account for difference in perfusion characteristics observed between adult and premature infant barriers [52] and account for the increased vulnerability of newborn brain to inflammation [53].

**Fig. 1:**
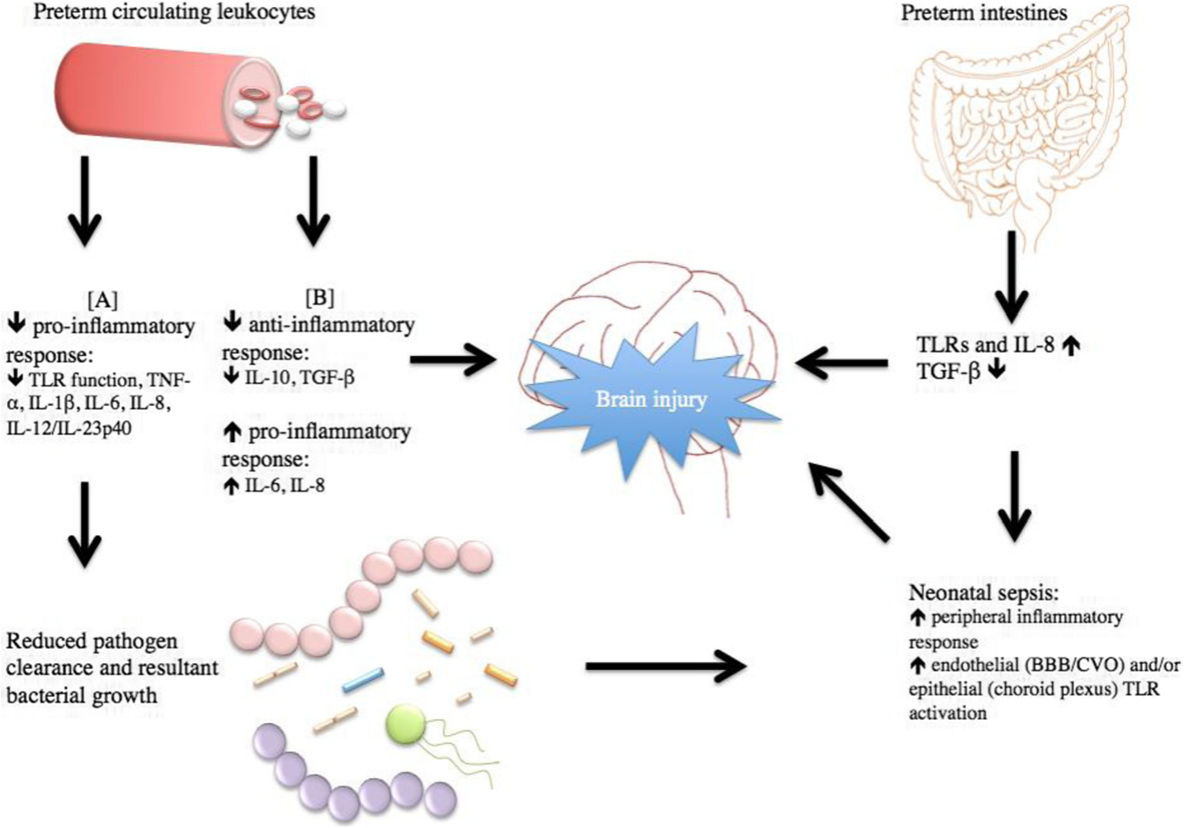
Immature immune response places preterm infants at higher risks of developing infection-induced sepsis that may lead to brain injury. **a** and **b** represent conflicting evidence. *TLR* toll-like receptor, *IL* interleukin, *TGF* transforming growth factor, *BBB* blood–brain barrier, *CVO* circumventricular organs

Inflammation in the periphery may cause sustained brain inflammation through an indirect mechanism (Fig. 1). Brain endothelial cells express toll-like receptors TLR-2, TLR3, TLR4, and TLR6 [54]. Circulating cells may express or release TLR ligands that interact with receptors present in brain endothelial cells that then activate downstream signaling to induce inflammation in the brain parenchyma. Indeed, TLR4 expression in CNS cells was required for mediating brain inflammation in response to systemic LPS in mice [55]. In addition, systemic IL-1β may activate receptors on the brain endothelium to induce prostaglandin E_2_ production in the brain which in turn mediates inflammation [56].

Alternatively, the circumventricular organs (CVO) (including the median eminence and adjacent neurohypophysis, the organum vasculosum lamina terminalis, the subfornical organ, and the area postrema [57]) and the choroid plexus have been suggested to serve as potential links between the CNS and peripheral blood-borne substances and for an alternative route for transfer of peripheral inflammatory signals to the brain [58–62].

Understanding such inflammatory mechanisms in the periphery is particularly helpful for purposes of finding specific biomarkers that can be targeted for easy diagnosis and effective therapeutic options. However, while cytokine detection in the periphery can have implications on inflammatory activities inside the brain, the detection window for such fluctuations in inflammatory cytokine levels may be narrow and unfortunately are not specific of inflammatory brain injury [39]. Understanding inflammatory mechanisms within the brain may provide a more specific target for finding the right biomarker.

### Mechanisms of brain injury

Brain injury observed in preterm neonates is likely multifactorial. However, inflammation, excitotoxicity, and immaturity of oligodendrocytes (OL) have been shown to play important contributory roles in impairing normal brain development. Microglial activation is at the cornerstone of inflammatory brain injury in the preterm infant. Implicated in a variety of developmental steps during brain maturation, microglial cells regroup in the periventricular zone, a main site with major axonal crossroads that corresponds to white matter areas that are most vulnerable in PVL, at the end of the second trimester [63, 64]. During the last trimester, microglia are able to mediate a M1 response characterized by the production of pro-inflammatory cytokines and chemokines, reactive oxygen and nitrogen species, excitatory amino acid, proteolytic enzymes, along with phagocytic activity [65–67]. Such pro-inflammatory response, suggesting M1 polarization of microglia, is commonly associated with white matter injury in response to cerebral insults [65, 68, 69]; the inhibition of such activation reduced the severity of lesions in neonatal animal models [70]. The ability to detect, in vivo, such shifts in microglia phenotype marked by cytokine/chemokine upregulation would provide highly meaningful diagnostic values for assessing brain injuries. As detailed in a later section, positron emission tomography (PET) may provide such values in the future. It is important to recognize that microglia also play a role in mediating elimination of excess axons and promoting neurogenesis and differentiation in the developing brain [67]. The neuroprotective role of M2 differentiation, associated with anti-inflammatory mechanisms, has been noted in adult animal models. M2 polarization is associated with the expression of neuroprotective cytokines such as TGF-β, IL-4, IL-10, and IL-13 [67]. Modulating the microglia towards an anti-inflammatory phenotype might be the key in reducing brain injury; obtaining tools to follow microglial activity noninvasively would be tremendously helpful.

Preterm infants have a high risk of developing white matter lesions in response to perinatal injuries and such injuries may be sensitized by previous exposure to inflammation [71–73]. Glutamate, an excitatory neurotransmitter in the brain, can mediate excitotoxicity through the activation of N-methyl-D-aspartate (NMDA), α-amino-3-hydroxy-5-methyl-4-isoxazolepropionic acid (AMPA), and kainate receptors [74]. Microglia activation can be attributed to NMDA receptor activation in newborn rodents through the use of selective receptor agonists [70]. While the expression of NMDA receptors has largely been investigated in the microglia, Manning et al. [75] have demonstrated that NMDA receptor expression is also detected in developing OLs in rats and humans. The expression of NMDA and AMPA receptors on developing OLs, during a period in the OL lineage with increased susceptibility, may contribute to white matter injuries seen in the developing brain [75]. It was generally accepted that premature OL (pre-OL) cell death due to vulnerability to oxidative stress and excitotoxicity contributed to the observed white matter loss [76]. However, emerging evidence has supported an alternative hypothesis that pre-OL maturational arrest may be responsible for the reduced myelination and subsequent white matter injury observed in animal studies [77, 78] and in human perinatal white matter injury [79]. The notion that failure of OL lineage differentiation, rather than cell death, mediates brain injury is supported by evidence of a proliferation of pre-OL pools following HI in white matter lesions [77]. Impaired myelination can be evaluated noninvasively with several tools using magnetic resonance imaging (MRI) as detailed in a later section. Visible quantifiable changes corresponding to mature myelinated fiber bundles can be evaluated using MRI starting at 37 weeks of gestation and be helpful to assess the impact of neuroprotective strategies.

### Biochemical biomarkers for brain injury

Intra-amniotic infections are associated with an increased risk of preterm delivery, which, in turn, may be associated with neurological sequelae in former preterm infants [80]. Microbial presence in the amniotic fluid may elicit maternal and fetal inflammatory response that are then responsible for neonatal complications. The association between elevated inflammatory cytokines IL-1β and IL-6 in the amniotic fluid and subsequent white matter injury has been noted in preterm infants [81]. Elevated levels of inflammatory cytokines in the cord blood including IL-1β, IL-6 IL-8, and TNF-α have also been shown to correlate with neonatal cerebral lesions as detected by MRI after parturition in human premature infants [82]. Furthermore, clinical evidence shows that elevated inflammatory response in the perinatal period has been demonstrated to correlate with long-term neonatal morbidities including cerebral palsy [83], psychomotor deficits [8], and non-neurological diseases including necrotizing enterocolitis (NEC) [84], bronchopulmonary dysplasia [85], and chronic lung disease [86, 87] in preterm neonates. However, as previously mentioned, antenatal exposure to infection may not necessarily be associated with an increased risk of adverse outcomes [11–14] and may even have a preconditioning effect [15, 16] since antenatal assessment of inflammation may be associated with a maternal response during pregnancy and may not truly reflect inflammatory response of the fetus. Dammann et al. [35] has suggested that cord blood and postnatal serum cytokine levels may reflect different waves of inflammatory responses in the fetal and neonatal circulation respectively and that cytokine levels in the blood may change drastically in the postnatal period. Nelson et al. [14] demonstrated that elevated inflammatory cytokines TNF-α, IL-1, IL-6, IL-8, along with interferon-γ (IFN-γ), vasoactive intestinal peptide, substance P, and calcitonin gene-related peptides in the neonatal blood correlated with PVL, ventriculomegaly, and severe germinal matric hemorrhage assessed by ultrasonography. Hecht et al. [88] also demonstrated that the elevation of several blood proteins, cytokines, and chemokine adhesion molecules was associated with white matter lesions. However, Kuban et al. [89] has noted that transient elevation of any single inflammation-associated cytokine in the blood did not predict cerebral palsy, but recurrent elevation within the first two postnatal weeks increased risk of adverse outcomes in preterm infants. Interestingly, risks of diparesis and hemiparesis were significantly increased when at least four inflammatory blood proteins were elevated during the first two postnatal weeks [89]. While these blood biomarkers may potentially predict brain lesions in the preterm infants, Ellison et al. [90] reported that that plasma levels of IL-6, IL-8, IL-10, TNF-α, and IFN-γ were not associated with cerebral spinal fluids of these cytokines nor did such plasma levels reflect brain injury as assessed by MRI. It is suggested, however, that cerebral spinal fluid (CSF) cytokine levels may be a more accurate predictor of cerebral white matter injury as preterm infants with white matter injury had higher CSF levels of IL-6, IL-10, and TNF-α. While elevation of various blood markers has been shown to be associated with white matter injury, these markers are diverse in classification and not necessarily predictive of white matter injury. Expression levels of many of these blood biomarkers are intertwined, thus making it difficult to pinpoint one biomarker that is specific for assessing brain injury. As mentioned previously, dysregulation of the immune system [40], causing an imbalance in the pro- and anti-inflammatory response, suggests that more than one biomarker may be involved in this process. In addition, the possibility of other organ injuries may interfere with blood assessments that reduce the accuracy in assessing brain injury based only on biomarkers in bodily fluids. Such biochemical assessments, however, may serve as useful screening tools for preterm infants at high risks of developing adverse neurological diseases. The emergence of the imaging techniques to assess brain injury has become prevalent in the clinic; the use of imaging biomarkers may provide a more accurate approach to assess inflammation/injury that is more specific to the brain.

### Ultrasonography

In humans, the clinical use of cranial ultrasound in detecting brain injury is the most prevalent technique used due to its relative safety, convenience of bedside scans, and cost-effectiveness that allows serial scanning of preterm infants at high risks [91] even if this technique is known for its notable limitations. It is able to detect ventriculomegaly, peri/intraventricular hemorrhage, cystic PVL [91, 92, 93], and cerebellar hemorrhages [91, 94]. However, cranial ultrasonography has reduced sensitivity for diffuse white matter lesions in the context of PVL whereas MRI and electroencephalography (EEG) may be more sensitive at detecting mild white matter injury [95–97] and with very little correlation with adverse neurological outcomes [98, 99]

In animals, high-resolution ultrasound is now possible within the first few days of birth in rodents, but this method quickly becomes limited due to skull calcification. Using alternative view angle in older animals offers better penetration without the need for scalp or skull removal [100]. Using a short laser pulse that induces a transient thermo-elastic expansion detected by the ultrasound receiver, photoacoustic tomography offers the ability to assess 2D coronal oxygen saturation maps. In an animal model of PVL, this method was able to detect a significant reduction in oxygen saturation in the corpus callosum, thus allowing for the monitoring of physiological changes noninvasively [101].

### Electroencephalography

Monitoring brain electrical activity can provide useful information to evaluate brain injury in preterm infants. EEG detects local field potentials produced by neurons that fire off electrical activity [102]. Both traditional EEG and amplitude-integrated EEG (aEEG), derived after processing from raw EEG recordings, provide valuable information with regard to monitoring seizures, brain maturation, and assessment of adverse neurological outcomes [103, 104].

#### In pre-clinical studies

**Fig. 2:**
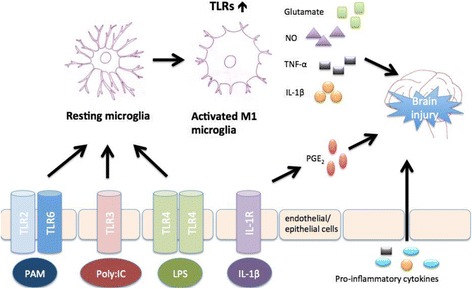
Proposed mechanisms mediating peripheral inflammation and brain injury. Sepsis can overstimulate TLRs on endothelial (BBB/CVO) and/or epithelial (choroid plexus) cells, which shift resting microglia to pro-inflammatory microglia that induce brain injury. *PAM* pathogen-associated molecules, *Poly:IC* polyinosinic: polycytidylic acid, *TLR* toll-like receptor, *LPS* lipopolysaccharide, *IL* interleukin, *IL-1R* interleukin-1 receptor, *TNF* tumor necrosis factor, *NO* nitric oxide, *PGE*_*2*_ prostaglandin E_2_

In a fetal sheep model of neuroinflammation [105], where a bolus intravenous LPS injection is administered directly to the fetus, typical hallmarks of white matter injury were detected by MRI together with an increased migroglia reaction (Fig. 2) and a reduction of OLs. In this study, the impact of inflammation was shown to be also associated with an impairment of a maturational EEG increase compared to the control group, indicative of reduced function of cortical neurons. In another fetal sheep model, continuous low-dose infusion of LPS produced increased the number of activated microglia and increased the number of TNF-α-positive cells in both the periventricular white matter and the cortex along with an impairment of normal EEG spectral edge frequency maturation that seemed to be associated with the observed inflammation in the LPS-exposed group [106].

#### In clinical studies

Wannabe et al. [107] suggests that serial recordings of EEG have prognostic values in detecting timing and mode of brain injuries in the preterm infants. A retrospective study by the group found that acute stage abnormalities, especially with EEG recordings performed within 2 days of birth, marked by various degrees of depression, strongly correlated with the severity of cerebral palsy [108]. Baud et al. [109], also in a retrospective study, suggested that the observation of positive rolandic sharpwaves in the rolandic regions (C3 and C4) within 7 days of birth correlated with severe PVL. Such waves are characterized by a sharp and transient wave of less than 500 ms in duration with a positive polarity. Okumura et al. [110] observed abnormal EEG patterns in preterm survivors with PVL in the early perinatal period where serial EEG recordings were marked by an increase in the number of frontal sharpwaves and occipital sharpwaves. It was observed that frontal sharpwaves and occipital sharpwaves were present when positive rolandic sharpwaves were not, indicating that the former two may be more sensitive markers for white matter injury and their sole presence may be useful in detecting less severe forms of PVL. The use of EEG as a simple diagnostic tool for detecting brain injuries has become popular in the recent years among clinicians. Although its use with respect to encephalopathy was mostly studied as a result of hypoxia-ischemia (HI), its prognostic value in predicting long-term outcomes has been noted [104, 111]. In a prospective study with 16 preterm infants [112], the presence of intraventricular hemorrhage and white matter damage was associated with prolonged interburst intervals and lower aEEG amplitudes.

### Magnetic resonance imaging

MRI and magnetic resonance spectroscopy (MRS) have become useful due to their high resolution and noninvasive means of monitoring macrostructural, microstructural, and metabolic developmental changes in the neonatal brain. Identifying ongoing brain injury in the setting of infection/inflammation will aid in recognizing newborns in need of targeted neuroprotection. To date, there is clinical evidence that MRI/MRS can identify early signs of injury along with a growing body of experimental data on animals to support such evidence.

#### Conventional imaging

Traditional magnetic resonance imaging, T1- and T2-weighted imaging, allow in vivo and ex vivo assessment of qualitative macrostructural changes in the brain and can detect overt injury [113].

**Fig. 3:**
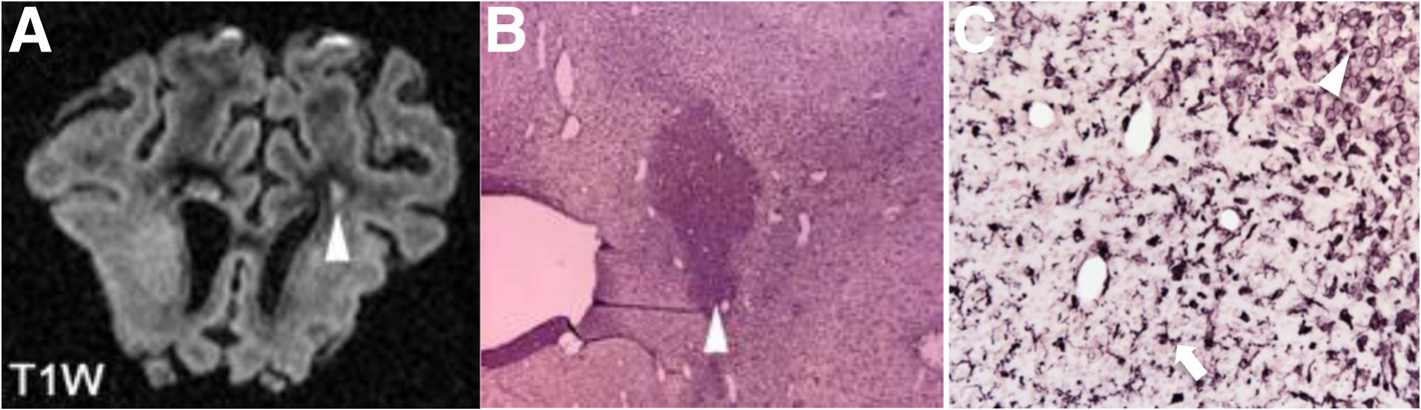
Coronal T1- and T2-weighted image with associated immuno-histochemistry of ex vivo fetal sheep brain exposed to lipopolysaccharide. **a** T1 hyperintensity (*arrowhead*) with **b** histological staining using ionized calcium binding adaptor molecule 1 (Iba1) in the corresponding area (*arrowhead*). **c** At higher magnification, the area shows dense macrophage-like microglia within the core of the periventricular white matter lesions (*arrowhead*). Microglia surrounding the core lesion demonstrates amoeboid/activated morphology (*black arrow*). The *dotted line* delineates the core of the lesion from the surrounding tissue. **a** and **b** are adapted from [105]

In pre-clinical studies, the ability to compare MRI data and histology provides insights into associated pathological changes following an inflammatory exposure (Fig. 3). Dean et al*.* [105] found that LPS exposure in fetal sheep induced cerebral injury, with T1 and T2 modifications seen 10 days after exposure, similar to what is seen in preterm infants with periventricular white matter injury. The distinguishing MRI features include localized T1 hyperintensities and diffuse T2 hyperintensities. The former corresponds to dense microglial activation, loss of neurofilaments, and accumulation of amyloid precursor protein. The latter corresponds to white matter rarefaction, diffuse activated microglia, increased apoptosis, and reduction of cells expressing oligodendrocyte transcription factor. In vivo assessment of signal changes in rat pups has shown, in the white matter, an increase in the T2 relaxation time constant, together with significant ventricular dilatation 4 days after LPS exposure [114]. However, if the assessment is performed immediately after an inflammatory exposure, conventional imaging may miss very early signs of inflammatory brain injury. In contrast, diffusion-weighted imaging and spectroscopy have shown extraordinary potential as detailed below.

In clinical studies, early conventional imaging can reveal more than periventricular cysts with early and sometime persistent periventricular lesions with localized T1 hyperintensities as seen in the sheep model exposed to LPS described earlier [115, 116]. In the setting of postnatal infection, conventional MRI has shown increased cerebellar hemorrhage [117].

#### Diffusion-weighted imaging

The apparent diffusion coefficient (ADC) is used as an assessment of the Brownian motion of water molecules. It is calculated from diffusion-weighted images. The restriction of water motion has been initially described in adult animal stroke models [118] with similar findings in neonatal studies [119, 120].

**Fig. 4:**
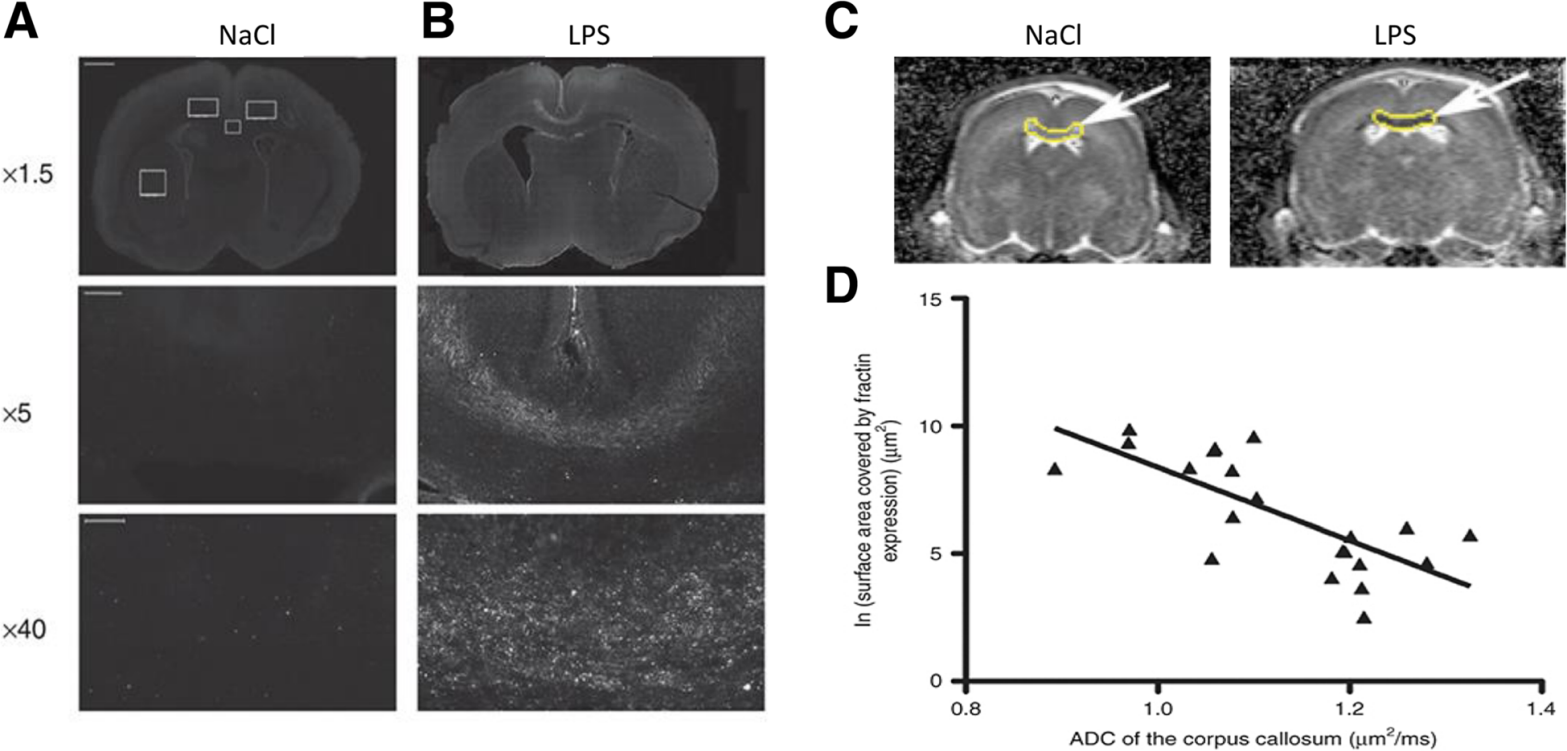
Fractin labeling for apoptosis. Immunolocalization of fractin (a specific apoptosis neoepitope derived from caspase-cleaved actin) in animals exposed to **a** 0.9 % sodium chloride (NaCl) or **b** lipopolysaccharide (LPS). Fractin labeling is intense in the corpus callosum of the LPS-treated animal. **c** Coronal apparent diffusion coefficient (ADC) maps of animals exposed to 0.9 % NaCl or LPS showing a significant restriction of diffusion in the corpus callosum (*highlighted*). **d** Anti-correlation between ADC and the natural logarithm of fractin expression in the corpus callosum. Adapted from [24]

In pre-clinical studies, the natural evolution of the ADC in the white matter has been quantified following LPS exposure [114]. Similar to HI in neonatal rats [120] and newborn term infants [121], the ADC has a biphasic profile with an initial restriction of diffusion in the white matter followed by an increase in diffusion 4 days after LPS exposure in neonatal rats [24]. The initial restriction of the ADC, measured 24 h after LPS exposure, was shown to be associated with apoptosis measured by fractin expression (Fig. 4a, b) [24]. Fractin is a caspase-specific cleavage product of actin that serves as a novel marker of apoptosis in brain injury. Yang et al. [122] demonstrated the presence of caspase-cleaved actin associated to degenerating neurons and plaque-associated microglia in Alzheimer’s disease and highlighted its role in pathological processes. The presence of an intense fractin immune-reactivity has been demonstrated in neuronal cell somas and dendrites [123]. In our study, there was a significant anti-correlation between the degree of ADC restriction and the natural logarithm of the fractin expression surface in the corpus callosum (Fig. 4c, d). The degree of ADC restriction, using noninvasive MRI, might serve as a potential marker to quantify significant injury in clinical settings and in animal experimentation. In human preterm infants during the first few weeks of life, such water diffusion restriction may be detected and was shown to predict severe cystic PVL [124, 125].

Few days following LPS exposure, the ADC gradually increases in animal studies. When compared to histology, the delayed increase in ADC has been associated with increase in edema, tissue rarefaction, decreased cell density, and increase in extracellular space [120, 126].

The radial diffusivity computed from diffusion tensor imaging (DTI) acquisition is traditionally related to myelin integrity as it measures diffusivity perpendicular to the axon. Increase in white matter radial diffusivity has been well described in mature animal model of multiple sclerosis and shown to be a reliable marker of dysmyelination [127]. However, the increase in radial diffusivity seen in LPS-exposed animals at day 4 following LPS injection was not attributed to myelination reduction but rather to decreased cell density [114]. Similarly, newborn mice exposed to IL-1β postnatally for 5 days and assessed 30 days later had also an increased in radial diffusivity. Although, there were fewer myelinated axons and the myelinated ones had a normal myelin sheath in contrast to findings described in adult animals [128]. Consequently, radial diffusivity is clearly affected by inflammatory injury but has to be interpreted in the context of the developing brain.

In humans, the delayed increased in ADC of the white matter has been shown in preterm infants imaged at term equivalent age. Counsell et al. [129] and Maalouf et al*.* [130] have demonstrated that, for preterm infants at term equivalent age with overt white matter injury and diffuse excessive high signal intensity on T2-weighted images, the ADC values in the white matter regions in the brain were significantly higher than for infants with intact white matter. In the setting of post-natal infection in preterm infants, diffusion imaging has revealed a similar delayed increase in mean diffusivity and increased radial diffusivity [117].

#### Tract-based spatial statistics

The development of user-independent approaches to quantify the impact of therapy is essential. Recently, tract-based spatial statistics (TBSS) has been identified as a potential tool in this regard. Designed to study fractional anisotropy maps compiled from DTI, TBSS is an approach that evaluates microarchitectural differences between groups in major fiber bundles. As the technique evaluates diffusion characteristics of major fiber bundles, it is not used as a specific tool to assess brain inflammation. TBSS is currently used in a prospective trial to test the efficiency of melatonin to protect the preterm brain (see below).

#### Spectroscopy

Proton MRS (^1^H-MRS) may be used to investigate brain metabolism and monitor development, which is characterized by a progressive increase in N-acetylaspartate (NAA) (a marker of neuronal integrity and maturation) and *myo*-inositol (a putative glial marker that also plays a role in the neuronal signaling of the phosphoinositide pathway) [131, 132]. An increased lactate level is indicative of anaerobic metabolism and mitochondrial dysfunction and was also described in microglial activation [132, 133].

In human neonates, brain injury was shown to alter the concentrations of NAA and lactate metabolites. In response to HI, NAA/choline ratio was shown to decrease [134] while lactate/choline ratio was shown to increase [135]. In preterm infants exposed to infection, a similar reduction in NAA/choline ratio was shown [117].

In animal models of LPS-induced brain inflammation, using very high magnetic field and short echo time, quantifiable increase in lactate concentration using the LCModel was also observed along with the appearance of lipid 13 [24], a peak corresponding to the methylene group (−CH_2_–) which is known to increase with apoptosis.

The impact of inflammation in the brain can be monitored in newborns and in equivalent animal models using MRI/MRS/DTI. As there are to date no blood biomarkers of cerebral integrity, the assessment of available imaging biomarkers is essential for monitoring brain injury and responses to therapy, but potentials and limitations of such approaches need to be better delineated.

#### Placenta MRI

##### In pre-clinical studies

As detailed earlier, pre-natal inflammatory exposure can modify the trajectory of brain development in preterm infants. A growing research domain of placental imaging may provide indications about underlying disease mechanisms and potentially serve as a useful biomarker. Pre-clinical studies have shown that in a murine model of placental inflammation, induced by systemic LPS administration, a reduction of T2-weighted intensity could be detected as early as 3–6 and 12 h after LPS exposure followed by a reduced placental perfusion seen by dynamic contrast-enhanced (DCE) T1-weighted imaging 12 h after LPS exposure [136]. In a landmark study using a rabbit model of placental uterine ischemia, DCE MRI was shown to correlate strongly with perfusion assessment using fluorescent microspheres but unfortunately systematically underestimated placental perfusion [137].

##### In clinical studies

Using conventional imaging, placental hemorrhages and ischemic lesions were detected by T1- and T2-weighted imaging but not chorioamnionitis [138]. In a case–control study using diffusion-weighted imaging, Sohlberg et al. extracted placental perfusion fraction and showed a strong correlation with ultrasound estimates [139]. The various aforementioned MRI techniques show the potential to provide insights for assessing placental complications. While evidence has shown that complications during pregnancy may provide both preconditioning and damaging sequelae to the fetal and neonatal brain, advancement in in vivo assessments during pregnancy may help to differentiate the two conditions.

### Positron emission tomography

PET allows for a noninvasive evaluation of microglia activation in the diseased brain. The technique takes advantage of the fact that the peripheral benzodiazepine receptor is primarily found on activated microglia and such receptors are capable of binding to radioactive ligands, which are in turn detectable using PET [140]. Hannestad et al. [141] found an increase in peripheral benzodiazepine receptor binding in baboon brains 1 and 4 h after receiving intravenous LPS in vivo. In addition to allowing for an evaluation of the timing of the inflammatory response, regional distribution of microglial activation was also computed in this particular study. However, to date, such studies have not been performed on human neonates and may be restricted to animal research for concept development.

### Therapeutic options

#### Pre-clinical studies

The role of IL-1β as a pro-inflammatory cytokine involved in eliciting injury in the immature brain has been well established through animal models [24, 25, 128, 142]. Thus, the therapeutic potential of IL-1 receptor antagonist has been explored. Girard et al. [142] demonstrated that postnatal administration of IL-1 receptor antagonist was successful in improving motor and behavioral outcomes after prenatal exposure to LPS along with reduced perinatal brain injury in rats. The use of IL-1 receptor antagonist has not been evaluated in preterm infants but has been evaluated in adult humans with severe sepsis; however, results were not promising with no statistically significant reduction of mortality [143]. Adult cerebral parenchyma has a different vulnerability compared to the newborn and, unfortunately, neuroprotection was not evaluated in this study.

The neuroprotective properties of IL-10 have also been demonstrated in premature animal models [144, 145]. Exogenous administration of IL-10 following LPS exposure has been known to reduce sensorimotor development impairment in the neonatal rat model [146] and offer neuroprotection by reducing brain injury in neonatal mice model [147]. IL-10 pretreatment also reduced ibotenate-induced excitotoxic brain injury [148]. Furthermore, in neonatal rats exposed to maternal LPS, IL-10 co-administered seemed to reduce white matter injury in the neonatal rats [18, 149]. Thus exogenous IL-10 might counteract the natural pattern of neonatal microglial activation towards a M1 response.

Minocycline, a second-generation derivative of tetracycline, has demonstrated anti-inflammatory and neuroprotective properties through suppressing microglial activation, reducing white matter injury [150–152] and improving neurobehavioral outcomes in premature animal models [153, 154]. In co-cultured microglia and neurons from human fetal brains, minocycline inhibited microglial activation and decreased cell death upon LPS exposure [155].

Melatonin is a powerful endogenous antioxidant with a high level of biosafety. Its efficacy in alleviating brain injury through promoting myelination, reducing inflammation, and reducing cell death has been demonstrated in various premature animal models [156–159]. Furthermore, the anti-oxidative and anti-inflammatory properties of melatonin in various neonatal animal models of NEC have shown to be effective in prevention [160, 161].

The neuroprotective properties of erythropoietin, a hematopoietic cytokine, have also been explored in the neonatal animal models where it was observed to attenuate microglial activation, reduce apoptosis, reduce OL damage, and promote neurogenesis and repair mechanisms [162–165].

Translating these strategies in human preterm infants is challenging and in contrast to animal studies, true neuroprotection assessment is complicated by the fact that detailed tissue analysis is not available and that accurate clinical evaluation can only be performed several years later.

#### Clinical studies

In clinical trials, the administration of melatonin has been shown to improve survival outcomes in septic term neonates [166]. A neuroprotective trial testing melatonin has been completed where TBSS was used as the main approach to assess its effect [167]. Unfortunately, preliminary results have not been conclusive [168]. This raises the question whether melatonin at the given dosage was unsuccessful or if TBSS is a sufficiently mature approach to evaluate the therapeutic response in these settings. As mentioned earlier, TBSS is designed to test only major fiber bundles, thus there is likelihood that a positive effect in smaller bundles might be overlooked.

Recently, in a Swiss multicenter research trial, erythropoietin has neuroprotective properties in the preterm babies. Leuchter et al*.* [169] showed that early high-dose treatment of erythropoietin in preterm infants significantly reduced the risk of white matter injury assessed at term equivalent age using a semi-quantitative approach on conventional MRI. Using a TBSS approach in the same cohort, erythropoietin was found to increase fractional anisotropy in major fiber bundles, a sign of increased maturation [170].

Corticosteroids play an immunomodulatory role by reducing the inflammatory response . However, clinical studies with early administration of corticosteroids as treatment for chronic lung disease in premature infants, while showed short-term benefits in improved ventilation, did not translate into long-term benefits [171]. In preterm infants, the administration of dexamethasone for the treatment of lung diseases has been shown to produce long-term complications including cerebral palsy [172], reduced cortical volume [173], and neurodevelopmental impairments or death [174]. Hydrocortisone, on the other hand, administered to premature infants in the early postnatal period did not seem to be associated with such long-term neurodevelopmental impairment and cerebral palsy [175–177] and may serve as a better alternative. Recently, in extremely preterm infants, an early and small dose of hydrocortisone was shown to be safe and reduced the risk of chronic lung disease, death, and patent ductus arteriosus [178]. Nonetheless, further research is required to identify its potential benefits in the setting of ongoing brain inflammatory injury.

## Conclusions

The etiology of white matter lesions seen in preterm infants is complex; various factors may predispose the immature brain to such injuries. The activation of the immune system is the body’s natural defense mechanism in fighting off invasive pathogens. However, uncontrolled response due to recurrent or severe infection may produce deleterious consequences including multi-organ failure, brain injury, and increased mortality. Various biochemical biomarkers have been shown to associate with brain injury in the preterm infants; however, there has yet emerged a single robust blood biomarker that is employed in clinical settings today. The emergence of imaging biomarkers as a noninvasive and sensitive diagnostic tool for early detection of cerebral white matter injury is gaining acceptance. Pharmacological interventions in the form of synthetic drugs, recombinant immunomodulators, and hormones have demonstrated anti-inflammatory/protective effects in animal models, however, with few promising candidates that have progressed to clinical trials. Further research is still required to come up with reliable tools to quantify noninvasively inflammation and monitor therapeutic response.

## Abbreviations

PVL: periventricular leukomalacia
LPS: lipopolysaccharide
CNS: central nervous system
BBB: blood–brain barrier
TNF-α: tumor necrosis factor alpha
IL: interleukin
TLR: toll-like receptors
CVO: circumventricular organs
OL: oligodendrocyte
PET: positron emission tomography
TGF-β: transforming growth factor-beta
NMDA: N-methyl-D-aspartate
AMPA: α-amino-3-hydroxy-5-methyl-4-isoxazolepropionic acid
Pre-OL: premature oligodendrocyte
MRI: magnetic resonance imaging
NEC: necrotizing enterocolitis
IFN-γ: interferon-gamma
CSF: cerebral spinal fluid
EEG: electroencephalography
aEEG: amplitude-integrated electroencephalography
HI: hypoxia-ischemia
MRS: magnetic resonance spectroscopy
DTI: diffusion tensor imaging
TBSS: tract-based spatial statistics
H-MRS: proton magnetic resonance spectroscopy
NAA: N-acetylaspartate

## References

[1] Virchow R (1867). Congenitale encephalitis und myelitis. Archiv f pathol Anat.

[2] Banker BQ, Larroche JC (1962). A form of neonatal anoxic encephalopathy. Arch Neurol.

[3] Volpe JJ (2005). Encephalopathy of prematurity includes neuronal abnormalities. Pediatrics.

[4] Volpe JJ (2009). The encephalopathy of prematurity—brain injury and impaired brain development inextricably intertwined. Sem Pediatr Neurol.

[5] Wu YW, Colford JM (2000). Chorioamnionitis as a risk factor for cerebral palsy: a meta-analysis. JAMA Pediatr.

[6] Costantine MM, How HY, Coppage K, Maxwell RA, Sibai BM (2007). Does peripartum infection increase the incidence of cerebral palsy in extremely low birthweight infants?. Am J Obstetr Gynecol.

[7] Zanardo V, Vedovato S, Suppiej A, Trevisanuto D, Migliore M, Di Venosa B, Chiarelli S (2008). Histological inflammatory responses in the placenta and early neonatal brain injury. Pediatr Dev Pathol.

[8] Hansen-Pupp I, Hallin A-L, Hellström-Westas L, Cilio C, Berg A-C, Stjernqvist K, Fellman V, Ley D (2008). Inflammation at birth is associated with subnormal development in very preterm infants. Pediatr Res.

[9] Suppiej A, Franzoi M, Vedovato S, Marucco A, Chiarelli S, Zanardo V (2009). Neurodevelopmental outcome in preterm histological chorioamnionitis. Early Hum Dev.

[10] Leviton A, Allred EN, Kuban KCK, Hecht JL, Onderdonk AB, O’Shea TM, Paneth N (2010). Microbiologic and histologic characteristics of the extremely preterm infant’s placenta predict white matter damage and later cerebral palsy. The ELGAN study. Pediatr Res.

[11] Andrews WW, Cliver SP, Biasini F, Peralta-Carcelen AM, Rector R, Alriksson-Schmidt AI, Faye-Petersen O, Carlo W, Goldenberg R, Hauth JC (2008). Early preterm birth: association between in utero exposure to acute inflammation and severe neurodevelopmental disability at 6 years of age. Am J Obstetr Gynecol.

[12] Chau V, Poskitt KJ, McFadden DE, Bowen-Roberts T, Synnes A, Brant R, Sargent MA, Soulikias W, Miller SP (2009). Effect of chorioamnionitis on brain development and injury in premature newborns. Ann Neurol.

[13] Reiman M, Kujari H, Maunu J, Parkkola R, Rikalainen H, Lapinleimu H, Lehtonen L, Haataja L (2008). Does placental inflammation relate to brain lesions and volume in preterm infants?. J Pediatr.

[14] Nelson KB, Grether JK, Dambrosia JM, Walsh E, Kohler S, Satyanarayana G, Nelson PG, Dickens BF, Phillips TM (2003). Neonatal cytokines and cerebral palsy in very preterm infants. Pediatr Res.

[15] Lahra MM, Jeffery HE (2004). A fetal response to chorioamnionitis is associated with early survival after preterm birth. Am J Obstetr Gynecol.

[16] Hendson L, Russell L, Robertson CMT, Liang Y, Chen Y, Abdalla A, Lacaze-Masmonteil T (2011). Neonatal and neurodevelopmental outcomes of very low birth weight infants with histologic chorioamnionitis. J Pediatr.

[17] Yoon BH, Kim CJ, Romero R, Jun JK, Park KH, Choi ST, Chi JG (1997). Experimentally induced intrauterine infection causes fetal brain white matter lesions in rabbits. Am J Obstetr Gynecol.

[18] Pang Y, Rodts-Palenik S, Cai Z, Bennett WA, Rhodes PG (2005). Suppression of glial activation is involved in the protection of IL-10 on maternal E. coli induced neonatal white matter injury. Brain Res Dev Brain Res.

[19] Poggi SH, Park J, Toso L, Abebe D, Roberson R, Woodard JE, Spong CY (2005). No phenotype associated with established lipopolysaccharide model for cerebral palsy. Am J Obstetr Gynecol.

[20] Nitsos I, Rees SM, Duncan J, Kramer BW, Harding R, Newnham JP, Moss TJM (2006). Chronic exposure to intra-amniotic lipopolysaccharide affects the ovine fetal brain. J Soc Gynecol Investig.

[21] Cai Z, Pan ZL, Pang Y, Evans OB, Rhodes PG (2000). Cytokine induction in fetal rat brains and brain injury in neonatal rats after maternal lipopolysaccharide administration. Pediatr Res.

[22] Hava G, Vered L, Yael M, Mordechai H, Mahoud H (2006). Alterations in behavior in adult offspring mice following maternal inflammation during pregnancy. Dev Psychobiol.

[23] Mallard C, Welin A-K, Peebles D, Hagberg H, Kjellmer I (2003). White matter injury following systemic endotoxemia or asphyxia in the fetal sheep. Neurochem Res.

[24] Lodygensky GA, Kunz N, Perroud E, Somm E, Mlynarik V, Hüppi PS, Gruetter R, Sizonenko SV (2013). Definition and quantification of acute inflammatory white matter injury in the immature brain by MRI/MRS at high magnetic field. Pediatr Res.

[25] Cai Z, Pang Y, Lin S, Rhodes PG (2003). Differential roles of tumor necrosis factor-α and interleukin-1 β in lipopolysaccharide-induced brain injury in the neonatal rat. Brain Res.

[26] Tissières P, Ochoda A, Dunn-Siegrist I, Drifte G, Morales M, Pfister R, Berner M, Pugin J (2012). Innate immune deficiency of extremely premature neonates can be reversed by interferon-γ. PLoS One.

[27] Melville JM, Moss TJM (2013). The immune consequences of preterm birth. Front Neurosci.

[28] Shane AL, Stoll BJ (2014). Neonatal sepsis: progress towards improved outcomes. J Infect.

[29] Lavoie PM, Huang Q, Jolette E, Whalen M, Nuyt AM, Audibert F, Speert DP, Lacaze-Masmonteil T, Soudeyns H, Kollmann TR (2010). Profound lack of interleukin (IL)-12/IL-23p40 in neonates born early in gestation is associated with an increased risk of sepsis. J Infect Dis.

[30] Strunk T, Currie A, Richmond P, Simmer K, Burgner D (2011). Innate immunity in human newborn infants: prematurity means more than immaturity. J Matern Fetal Neonatal Med.

[31] Carr R (2000). Neutrophil production and function in newborn infants. Br J Haematol.

[32] Strunk T, Prosser A, Levy O, Philbin V, Simmer K, Doherty D, Charles A, Richmond P, Burgner D, Currie A (2012). Responsiveness of human monocytes to the commensal bacterium Staphylococcus epidermidis develops late in gestation. Pediatr Res.

[33] Shen C-M, Lin S-C, Niu D-M, Kou YR (2013). Development of monocyte Toll-like receptor 2 and Toll-like receptor 4 in preterm newborns during the first few months of life. Pediatr Res.

[34] Schultz C, Rott C, Temming P, Schlenke P, Möller JC, Bucsky P (2002). Enhanced interleukin-6 and interleukin-8 synthesis in term and preterm infants. Pediatr Res.

[35] Dammann O, Phillips TM, Allred EN, O’Shea TM, Paneth N, Van Marter LJ, Bose C, Ehrenkranz RA, Bednarek FJ, Naples M, Leviton A, ELGAN study Investigators (2001). Mediators of fetal inflammation in extremely low gestational age newborns. Cytokine.

[36] Nanthakumar N, Meng D, Goldstein AM, Zhu W, Lu L, Uauy R, Llanos A, Claud EC, Walker WA (2011). The mechanism of excessive intestinal inflammation in necrotizing enterocolitis: an immature innate immune response. PLoS One.

[37] Adib-Conquy M, Cavaillon J-M (2009). Compensatory anti-inflammatory response syndrome. Thromb Haemost.

[38] Schultz C, Temming P, Bucsky P, Göpel W, Strunk T, Härtel C (2004). Immature anti-inflammatory response in neonates. Clin Exp Immunol.

[39] Maheshwari A, Schelonka RL, Dimmitt RA, Carlo WA, Munoz-Hernandez B, Das A, McDonald SA, Thorsen P, Skogstrand K, Hougaard DM, Higgins RD, for the Eunice Kennedy Shriver National Institute of Child Health, Network HDNR (2014). Cytokines associated with necrotizing enterocolitis in extremely-low-birth-weight infants. Pediatr Res.

[40] Chalak LF, Sánchez PJ, Adams-Huet B, Laptook AR, Heyne RJ, Rosenfeld CR (2014). Biomarkers for severity of neonatal hypoxic-ischemic encephalopathy and outcomes in newborns receiving hypothermia therapy. J Pediatr.

[41] Mallard C (2012). Innate immune regulation by toll-like receptors in the brain. ISRN Neurol.

[42] Gutierrez EG, Banks WA, Kastin AJ (1993). Murine tumor necrosis factor alpha is transported from blood to brain in the mouse. J Neuroimmunol.

[43] Banks WA, Kastin AJ, Gutierrez EG (1994). Penetration of interleukin-6 across the murine blood–brain barrier. Neurosci Lett.

[44] Leviton A (1993). Preterm birth and cerebral palsy: is tumor necrosis factor the missing link?. Dev Med Child Neurol.

[45] Banks WA, Ortiz L, Plotkin SR, Kastin AJ (1991). Human interleukin (IL) 1 alpha, murine IL-1 alpha and murine IL-1 beta are transported from blood to brain in the mouse by a shared saturable mechanism. J Pharmacol Exp Ther.

[46] Smith PLP, Hagberg H, Naylor AS, Mallard C (2014). Neonatal peripheral immune challenge activates microglia and inhibits neurogenesis in the developing murine hippocampus. Dev Neurosci.

[47] Banks WA, Robinson SM (2010). Minimal penetration of lipopolysaccharide across the murine blood–brain barrier. Brain Behav Immun.

[48] Singh AK, Jiang Y (2004). How does peripheral lipopolysaccharide induce gene expression in the brain of rats?. Toxicology.

[49] Tao-Cheng JH, Nagy Z, Brightman MW (1987). Tight junctions of brain endothelium in vitro are enhanced by astroglia. J Neurosci.

[50] Janzer RC, Raff MC (1987). Astrocytes induce blood–brain barrier properties in endothelial cells. Nature.

[51] Daneman R, Zhou L, Kebede AA, Barres BA (2010). Pericytes are required for blood–brain barrier integrity during embryogenesis. Nature.

[52] Ek CJ, Dziegielewska KM, Habgood MD, Saunders NR (2012). Barriers in the developing brain and neurotoxicology. Neurotoxicology.

[53] Stolp HB, Dziegielewska KM (2009). Review: role of developmental inflammation and blood–brain barrier dysfunction in neurodevelopmental and neurodegenerative diseases. Neuropathol Appl Neurobiol.

[54] Nagyőszi P, Wilhelm I, Farkas AE, Fazakas C, Dung NTK, Haskó J, Krizbai IA (2010). Expression and regulation of toll-like receptors in cerebral endothelial cells. Neurochem Int.

[55] Chakravarty S, Herkenham M (2005). Toll-like receptor 4 on nonhematopoietic cells sustains CNS inflammation during endotoxemia, independent of systemic cytokines. J Neurosci.

[56] Gosselin D, RIVEST S (2008). MyD88 signaling in brain endothelial cells is essential for the neuronal activity and glucocorticoid release during systemic inflammation. Mol Psychiatry.

[57] Ganong WF (2000). Circumventricular organs: definition and role in the regulation of endocrine and autonomic function. Clin Exp Pharmacol Physiol.

[58] LAFLAMME N, RIVEST S (2001). Toll-like receptor 4: the missing link of the cerebral innate immune response triggered by circulating gram-negative bacterial cell wall components. FASEB J.

[59] Dantzer R, O’Connor JC, Freund GG, Johnson RW, Kelley KW (2008). From inflammation to sickness and depression: when the immune system subjugates the brain. Nat Rev Neurosci.

[60] Brinker T, Stopa E, Morrison J (2014). A new look at cerebrospinal fluid circulation. Fluids Barriers CNS.

[61] Stridh L, Ek CJ, Wang X, Nilsson H, Mallard C (2013). Regulation of toll-like receptors in the choroid plexus in the immature brain after systemic inflammatory stimuli. Transl Stroke Res.

[62] D’angelo B, Ek CJ, Sandberg M, Mallard C (2013). Expression of the Nrf2-system at the blood-CSF barrier is modulated by neonatal inflammation and hypoxia-ischemia. J Inherit Metab Dis.

[63] Verney C, Pogledic I, Biran V, Adle-Biassette H, Fallet-Bianco C, Gressens P (2012). Microglial reaction in axonal crossroads is a hallmark of noncystic periventricular white matter injury in very preterm infants. J Neuropathol Exp Neurol.

[64] Baburamani AA, Supramaniam VG, Hagberg H, Mallard C (2014). Microglia toxicity in preterm brain injury. Reprod Toxicol.

[65] Thornton C, Rousset CI, Kichev A, Miyakuni Y, Vontell R, Baburamani AA, Fleiss B, Gressens P, Hagberg H (2012). Molecular mechanisms of neonatal brain injury. Neurol Res Int.

[66] Perry VH, Cunningham C, Holmes C (2007). Systemic infections and inflammation affect chronic neurodegeneration. Nat Rev Immunol.

[67] Czeh M, Gressens P, Kaindl AM (2011). The yin and yang of microglia. Dev Neurosci.

[68] Yao L, Kan EM, Lu J, Hao A, Dheen ST, Kaur C, Ling E-A (2013). Toll-like receptor 4 mediates microglial activation and production of inflammatory mediators in neonatal rat brain following hypoxia: role of TLR4 in hypoxic microglia. J Neuroinflammation.

[69] Ivacko JA, Sun R, Silverstein FS (1996). Hypoxic-ischemic brain injury induces an acute microglial reaction in perinatal rats. Pediatr Res.

[70] Dommergues M-A, Plaisant F, Verney C, Gressens P (2003). Early microglial activation following neonatal excitotoxic brain damage in mice: a potential target for neuroprotection. Neuroscience.

[71] Eklind S, Mallard C, Arvidsson P, Hagberg H (2005). Lipopolysaccharide induces both a primary and a secondary phase of sensitization in the developing rat brain. Pediatr Res.

[72] Hickey E, Shi H, Van Arsdell G, Askalan R (2011). Lipopolysaccharide-induced preconditioning against ischemic injury is associated with changes in toll-like receptor 4 expression in the rat developing brain. Pediatr Res.

[73] Destot-Wong K-D, Liang K, Gupta SK, Favrais G, Schwendimann L, Pansiot J, Baud O, Spedding M, Lelièvre V, Mani S, Gressens P (2009). The AMPA receptor positive allosteric modulator, S18986, is neuroprotective against neonatal excitotoxic and inflammatory brain damage through BDNF synthesis. Neuropharmacology.

[74] Wu X, Zhu D, Jiang X, Okagaki P, Mearow K, Zhu G, McCall S, Banaudha K, Lipsky RH, Marini AM (2004). AMPA protects cultured neurons against glutamate excitotoxicity through a phosphatidylinositol 3-kinase-dependent activation in extracellular signal-regulated kinase to upregulate BDNF gene expression. J Neurochem.

[75] Manning SM, Talos DM, Zhou C, Selip DB, Park H-K, Park C-J, Volpe JJ, Jensen FE (2008). NMDA receptor blockade with memantine attenuates white matter injury in a rat model of periventricular leukomalacia. J Neurosci.

[76] Khwaja O, Volpe JJ (2008). Pathogenesis of cerebral white matter injury of prematurity. Arch Dis Child Fetal Neonatal Ed.

[77] Segovia KN, McClure M, Moravec M, Luo NL, Wan Y, Gong X, Riddle A, Craig A, Struve J, Sherman LS, Back SA (2008). Arrested oligodendrocyte lineage maturation in chronic perinatal white matter injury. Ann Neurol.

[78] Riddle A, Dean J, Buser JR, Gong X, Maire J, Chen K, Ahmad T, Cai V, Nguyen T, Kroenke CD, Hohimer AR, Back SA (2011). Histopathological correlates of magnetic resonance imaging–defined chronic perinatal white matter injury. Ann Neurol.

[79] Buser JR, Maire J, Riddle A, Gong X, Nguyen T, Nelson K, Luo NL, Ren J, Struve J, Sherman LS, Miller SP, Chau V, Hendson G, Ballabh P, Grafe MR, Back SA (2012). Arrested preoligodendrocyte maturation contributes to myelination failure in premature infants. Ann Neurol.

[80] Goldenberg RL, Hauth JC, Andrews WW (2000). Intrauterine infection and preterm delivery. N Engl J Med.

[81] Yoon BH, Jun JK, Romero R, Park KH, Gomez R, Choi JH, Kim IO (1997). Amniotic fluid inflammatory cytokines (interleukin-6, interleukin-1beta, and tumor necrosis factor-alpha), neonatal brain white matter lesions, and cerebral palsy. Am J Obstetr Gynecol.

[82] Duggan PJ, Maalouf EF, Watts TL, Sullivan MH, Counsell SJ, Allsop J, Al-Nakib L, Rutherford MA, Battin M, Roberts I, Edwards AD (2001). Intrauterine T-cell activation and increased proinflammatory cytokine concentrations in preterm infants with cerebral lesions. Lancet.

[83] Yoon BH, Romero R, Park JS, Kim CJ, Kim SH, Choi JH, Han TR (2000). Fetal exposure to an intra-amniotic inflammation and the development of cerebral palsy at the age of three years. Am J Obstetr Gynecol.

[84] Satar M, Turhan E, Yapicioglu H, Narli N, Ozgunen FT, Cetiner S (2008). Cord blood cytokine levels in neonates born to mothers with prolonged premature rupture of membranes and its relationship with morbidity and mortality. Eur Cytokine Netw.

[85] Rocha G, Proença E, Guedes A, Carvalho C, Areias A, Ramos JP, Rodrigues T, Guimarães H (2012). Cord blood levels of IL-6, IL-8 and IL-10 may be early predictors of bronchopulmonary dysplasia in preterm newborns small for gestational age. Dis Markers.

[86] An H, Nishimaki S, Ohyama M, Haruki A, Naruto T, Kobayashi N, Sugai T, Kobayashi Y, Mori M, Seki K, Yokota S (2004). Interleukin-6, interleukin-8, and soluble tumor necrosis factor receptor-I in the cord blood as predictors of chronic lung disease in premature infants. Am J Obstetr Gynecol.

[87] Takao D, Ibara S, Tokuhisa T, Ishihara C, Maede Y, Matsui T, Tokumasu H, Sato K, Hirakawa E, Kabayama C, Yamamoto M (2014). Predicting onset of chronic lung disease using cord blood cytokines. Pediatr Int.

[88] Hecht JL, Fichorova RN, Tang VF, Allred EN, McElrath TF, Leviton A, ELGAN study Investigators (2011). Relationship between neonatal blood protein concentrations and placenta histologic characteristics in extremely low GA newborns. Pediatr Res.

[89] Kuban KCK, O’Shea TM, Allred EN, Paneth N, Hirtz D, Fichorova RN, Leviton A, ELGAN study Investigators (2014). Systemic inflammation and cerebral palsy risk in extremely preterm infants. J Child Neurol.

[90] Ellison VJ, Mocatta TJ, Winterbourn CC, Darlow BA, Volpe JJ, Inder TE (2005). The relationship of CSF and plasma cytokine levels to cerebral white matter injury in the premature newborn. Pediatr Res.

[91] Panigrahy A, Wisnowski JL, Furtado A, Lepore N, Paquette L, Bluml S (2012). Neuroimaging biomarkers of preterm brain injury: toward developing the preterm connectome. Pediatr Radiol.

[92] Maalouf EF, Duggan PJ, Counsell SJ, Rutherford MA, Cowan F, Azzopardi D, Edwards AD (2001). Comparison of findings on cranial ultrasound and magnetic resonance imaging in preterm infants. Pediatrics.

[93] O’Shea TM, Counsell SJ, Bartels DB, Dammann O (2005). Magnetic resonance and ultrasound brain imaging in preterm infants. Early Hum Dev.

[94] van Wezel-Meijler G, Steggerda SJ, Leijser LM (2010). Cranial ultrasonography in neonates: role and limitations. Semin Perinatol.

[95] Miller SP, Cozzio CC, Goldstein RB, Ferriero DM, Partridge JC, Vigneron DB, Barkovich AJ (2003). Comparing the diagnosis of white matter injury in premature newborns with serial MR Imaging and transfontanel ultrasonography findings. AJNR Am J Neuroradiol.

[96] Inder TE, Anderson NJ, Spencer C, Wells S, Volpe JJ (2003). White matter injury in the premature infant: a comparison between serial cranial sonographic and MR findings at term. AJNR Am J Neuroradiol.

[97] Ciambra G, Arachi S, Protano C, Cellitti R, Caoci S, Di Biasi C, Gualdi G, De Curtis M (2013). Accuracy of transcranial ultrasound in the detection of mild white matter lesions in newborns. Neuroradiol J.

[98] Mirmiran M, Barnes PD, Keller K, Constantinou JC, Fleisher BE, Hintz SR, Ariagno RL (2004). Neonatal brain magnetic resonance imaging before discharge is better than serial cranial ultrasound in predicting cerebral palsy in very low birth weight preterm infants. Pediatrics.

[99] Woodward LJ, Anderson PJ, Austin NC, Howard K, Inder TE (2006). Neonatal MRI to predict neurodevelopmental outcomes in preterm infants. N Engl J Med.

[100] Giustetto P, Filippi M, Castano M, Terreno E. Non-invasive parenchymal, vascular and metabolic high-frequency ultrasound and photoacoustic rat deep brain imaging. J Vis Exp 2015. doi: 10.3791/52162 10.3791/52162 PMC4401174 25867127

[101] Guevara E, Berti R, Londono I, Xie N, Bellec P, Lesage F, Lodygensky GA (2013). Imaging of an inflammatory injury in the newborn rat brain with photoacoustic tomography. PLoS One.

[102] Mento G, Bisiacchi PS (2012). Neurocognitive development in preterm infants: insights from different approaches. Neurosci Biobehav Rev.

[103] Shany E, Berger I (2011). Neonatal electroencephalography: review of a practical approach. J Child Neurol.

[104] El-Dib M, Chang T, Tsuchida TN, Clancy RR (2009). Amplitude-integrated electroencephalography in neonates. Pediatr Neurol.

[105] Dean JM, van de Looij Y, Sizonenko SV, Lodygensky GA, Lazeyras F, Bolouri H, Kjellmer I, Hüppi PS, Hagberg H, Mallard C (2011). Delayed cortical impairment following lipopolysaccharide exposure in preterm fetal sheep. Ann Neurol.

[106] Keogh MJ, Bennet L, Drury PP, Booth LC, Mathai S, Naylor AS, Fraser M, Gunn AJ (2012). Subclinical exposure to low-dose endotoxin impairs EEG maturation in preterm fetal sheep. Am J Physiol Regul Integr Comp Physiol.

[107] Watanabe K, Hayakawa F, Okumura A (1999). Neonatal EEG: a powerful tool in the assessment of brain damage in preterm infants. Brain and Development.

[108] Maruyama K, Okumura A, Hayakawa F, Kato T (2002). Prognostic value of EEG depression in preterm infants for later development of cerebral palsy. Neuropediatrics.

[109] Baud O, d’Allest A-M, Lacaze-Masmonteil T, Zupan V, Nedelcoux H, Boithias C, Delaveaucoupet J, Dehan M (1998). The early diagnosis of periventricular leukomalacia in premature infants with positive rolandic sharp waves on serial electroencephalography. The J Pediatr.

[110] Okumura A, Hayakawa F, Kato T, Maruyama K, Kubota T, Suzuki M, Kidokoro H, Kuno K, Watanabe K (2003). Abnormal sharp transients on electroencephalograms in preterm infants with periventricular leukomalacia. The J Pediatr.

[111] Shah DK, de Vries LS, Hellström-Westas L, Toet MC, Inder TE (2008). Amplitude-integrated electroencephalography in the newborn: a valuable tool. Pediatrics.

[112] Wikström S, Ley D, Hansen-Pupp I, Rosén I, Hellström-Westas L (2008). Early amplitude-integrated EEG correlates with cord TNF-α and brain injury in very preterm infants. Acta Paediatr.

[113] Lodygensky GA, Vasung L, Sizonenko SV, Hüppi PS (2010). Neuroimaging of cortical development and brain connectivity in human newborns and animal models. J Anat.

[114] Lodygensky GA, West T, Stump M, Holtzman DM, Inder TE, Neil JJ (2010). In vivo MRI analysis of an inflammatory injury in the developing brain. Brain Behav Immun.

[115] Nanba Y, Matsui K, Aida N, Sato Y, Toyoshima K, Kawataki M, Hoshino R, Ohyama M, Itani Y, Goto A, Oka A (2007). Magnetic resonance imaging regional T1 abnormalities at term accurately predict motor outcome in preterm infants. Pediatrics.

[116] Miller SP, Ferriero DM, Leonard C, Piecuch R, Glidden DV, Partridge JC, Perez M, Mukherjee P, Vigneron DB, Barkovich AJ (2005). Early brain injury in premature newborns detected with magnetic resonance imaging is associated with adverse early neurodevelopmental outcome. The J Pediatr.

[117] Chau V, Brant R, Poskitt KJ, Tam EWY, Synnes A, Miller SP (2012). Postnatal infection is associated with widespread abnormalities of brain development in premature newborns. Pediatr Res.

[118] Norris DG, Niendorf T, Hoehn-Berlage M, Kohno K, Schneider EJ, Hainz P, Hropot M, Leibfritz D (1994). Incidence of apparent restricted diffusion in three different models of cerebral infarction. Magn Reson Imaging.

[119] Tuor UI, Kozlowski P, Del Bigio MR, Ramjiawan B, Su S, Malisza K, Saunders JK (1998). Diffusion- and T2-weighted increases in magnetic resonance images of immature brain during hypoxia-ischemia: transient reversal posthypoxia. Exp Neurol.

[120] Nedelcu J, Klein MA, Aguzzi A, Boesiger P, Martin E (1999). Biphasic edema after hypoxic-ischemic brain injury in neonatal rats reflects early neuronal and late glial damage. Pediatr Res.

[121] McKinstry RC, Miller JH, Snyder AZ, Mathur A, Schefft GL, Almli CR, Shimony JS, Shiran SI, Neil JJ (2002). A prospective, longitudinal diffusion tensor imaging study of brain injury in newborns. Neurology.

[122] Yang F, Sun X, Beech W, Teter B, Wu S, Sigel J, Vinters HV, Frautschy SA, Cole GM (1998). Antibody to caspase-cleaved actin detects apoptosis in differentiated neuroblastoma and plaque-associated neurons and microglia in Alzheimer’s disease. Am J Pathol.

[123] Hendrickson ML, Ling C, Kalil RE (2012). Degeneration of axotomized projection neurons in the rat dLGN: temporal progression of events and their mitigation by a single administration of FGF2. PLoS One.

[124] Lodygensky GA, Menache C, Hüppi PS, Perlman J (2011). Magnetic resonance imaging’s role in the care of the infant at risk for brain injury. Neurology: neonatology questions and controversies.

[125] Inder T, Hüppi PS, Zientara GP, Maier SE, Jolesz FA, di Salvo D, Robertson R, Barnes PD, Volpe JJ (1999). Early detection of periventricular leukomalacia by diffusion-weighted magnetic resonance imaging techniques. J Pediatr.

[126] Lodygensky GA, Marques JP, Maddage R, Perroud E, Sizonenko SV, Hüppi PS, Gruetter R (2012). In vivo assessment of myelination by phase imaging at high magnetic field. Neuroimage.

[127] Song AW (2012). Diffusion modulation of the fMRI signal: early investigations on the origin of the BOLD signal. Neuroimage.

[128] Favrais G, van de Looij Y, Fleiss B, Ramanantsoa N, Bonnin P, Stoltenburg Didinger G, Lacaud A, Saliba E, Dammann O, Gallego J, Sizonenko S, Hagberg H, Lelièvre V, Gressens P (2011). Systemic inflammation disrupts the developmental program of white matter. Ann Neurol.

[129] Counsell SJ, Allsop JM, Harrison MC, Larkman DJ, Kennea NL, Kapellou O, Cowan FM, Hajnal JV, Edwards AD, Rutherford MA (2003). Diffusion-weighted imaging of the brain in preterm infants with focal and diffuse white matter abnormality. Pediatrics.

[130] Maalouf EF, Duggan PJ, Rutherford MA, Counsell SJ, Fletcher AM, Battin M, Cowan F, Edwards AD (1999). Magnetic resonance imaging of the brain in a cohort of extremely preterm infants. J Pediatr.

[131] Tkác I, Rao R, Georgieff MK, Gruetter R (2003). Developmental and regional changes in the neurochemical profile of the rat brain determined by in vivo 1H NMR spectroscopy. Magn Reson Med.

[132] Lodygensky GA, Inder TE, Neil JJ (2008). Application of magnetic resonance imaging in animal models of perinatal hypoxic-ischemic cerebral injury. Int J Dev Neurosci.

[133] López-Villegas D, Lenkinski RE, Wehrli SL, Ho WZ, Douglas SD (1995). Lactate production by human monocytes/macrophages determined by proton MR spectroscopy. Magn Reson Med.

[134] Groenendaal F, Veenhoven RH, van der Grond J, Jansen GH, Witkamp TD, de Vries LS (1994). Cerebral lactate and N-acetyl-aspartate/choline ratios in asphyxiated full-term neonates demonstrated in vivo using proton magnetic resonance spectroscopy. Pediatr Res.

[135] Barkovich AJ, Baranski K, Vigneron D, Partridge JC, Hallam DK, Hajnal BL, Ferriero DM (1999). Proton MR spectroscopy for the evaluation of brain injury in asphyxiated, term neonates. AJNR Am J Neuroradiol.

[136] Girard S, Tremblay L, Lepage M, Sébire G (2012). Early detection of placental inflammation by MRI enabling protection by clinically relevant IL-1Ra administration. Am J Obstetr Gynecol.

[137] Drobyshevsky A, Prasad PV. Placental perfusion in uterine ischemia model as evaluated by dynamic contrast enhanced MRI. J Magn Reson Imaging. 2015:n/a–n/a 10.1002/jmri.24830 PMC5558458 25854322

[138] Linduska N, Dekan S, Messerschmidt A, Kasprian G, Brugger PC, Chalubinski K, Weber M, Prayer D (2009). Placental pathologies in fetal MRI with pathohistological correlation. Placenta.

[139] Sohlberg S, Mulic-Lutvica A, Olovsson M, Weis J, Axelsson O, Wikström J, Wikström A-K. MRI estimated placental perfusion in fetal growth assessment. Ultrasound Obstet Gynecol. 2015:n/a–n/a. 10.1002/uog.14786 PMC5063104 25640054

[140] Cagnin A, Kassiou M, Meikle SR, Banati RB (2007). Positron emission tomography imaging of neuroinflammation. Neurotherapeutics.

[141] Hannestad J, Gallezot J-D, Schafbauer T, Lim K, Kloczynski T, Morris ED, Carson RE, Ding Y-S, Cosgrove KP (2012). Endotoxin-induced systemic inflammation activates microglia: [^11^C]PBR28 positron emission tomography in nonhuman primates. Neuroimage.

[142] Girard S, Sébire H, Brochu M-E, Briota S, Sarret P, Sébire G (2012). Postnatal administration of IL-1Ra exerts neuroprotective effects following perinatal inflammation and/or hypoxic-ischemic injuries. Brain Behav Immun.

[143] Opal SM, Fisher CJ, Dhainaut J-FA, Vincent J-L, Brase R, Lowry SF, Sadoff JC, Slotman GJ, Levy H, Balk RA, Shelly MP, Pribble JP, LaBrecque JF, Lookabaugh J, Donovan H, Dubin H, Baughman R, Norman J, DeMaria E, Matzel K, Abraham E, Seneff M (1997). Confirmatory interleukin-1 receptor antagonist trial in severe sepsis: a phase III, randomized, doubleblind, placebo-controlled, multicenter trial. Crit Care Med.

[144] Li S-J, Liu W, Wang J-L, Zhang Y, Zhao D-J, Wang T-J, Li Y-Y (2014). The role of TNF-α, IL-6, IL-10, and GDNF in neuronal apoptosis in neonatal rat with hypoxic-ischemic encephalopathy. Eur Rev Med Pharmacol Sci.

[145] Gonzalez P, Burgaya F, Acarin L, Peluffo H, Castellano B, Gonzalez B (2009). Interleukin-10 and interleukin-10 receptor-I are upregulated in glial cells after an excitotoxic injury to the postnatal rat brain. J Neuropathol Exp Neurol.

[146] Wallace KL, Lopez J, Shaffery JP, Wells A, Paul IA, Bennett WA (2010). Interleukin-10/Ceftriaxone prevents E. coli-induced delays in sensorimotor task learning and spatial memory in neonatal and adult Sprague–Dawley rats. Brain Res Bull.

[147] Mittal R, Gonzalez-Gomez I, Panigrahy A, Goth K, Bonnet R, Prasadarao NV (2010). IL-10 administration reduces PGE-2 levels and promotes CR3-mediated clearance of Escherichia coli K1 by phagocytes in meningitis. J Exp Med.

[148] Mesples B, Plaisant F, Gressens P (2003). Effects of interleukin-10 on neonatal excitotoxic brain lesions in mice. Brain Res Dev Brain Res.

[149] Rodts-Palenik S, Wyatt-Ashmead J, Pang Y, Thigpen B, Cai Z, Rhodes P, Martin JN, Granger J, Bennett WA (2004). Maternal infection-induced white matter injury is reduced by treatment with interleukin-10. Am J Obstetr Gynecol.

[150] Lechpammer M, Manning SM, Samonte F, Nelligan J, Sabo E, Talos DM, Volpe JJ, Jensen FE (2008). Minocycline treatment following hypoxic/ischaemic injury attenuates white matter injury in a rodent model of periventricular leucomalacia. Neuropathol Appl Neurobiol.

[151] Cai Z, Lin S, Fan L-W, Pang Y, Rhodes PG (2006). Minocycline alleviates hypoxic–ischemic injury to developing oligodendrocytes in the neonatal rat brain. Neuroscience.

[152] Fan L-W, Pang Y, Lin S, Rhodes PG, Cai Z (2005). Minocycline attenuates lipopolysaccharide-induced white matter injury in the neonatal rat brain. Neuroscience.

[153] Zhu F, Zheng Y, Ding Y-Q, Liu Y, Zhang X, Wu R, Guo X, Zhao J (2014). Minocycline and risperidone prevent microglia activation and rescue behavioral deficits induced by neonatal intrahippocampal injection of lipopolysaccharide in rats. PLoS One.

[154] Fan LW, Pang Y, Lin S, Tien LT, Ma T, Rhodes PG, Cai Z (2005). Minocycline reduces lipopolysaccharide-induced neurological dysfunction and brain injury in the neonatal rat. J Neurosci Res.

[155] Filipovic R, Zecevic N (2008). Neuroprotective role of minocycline in co-cultures of human fetal neurons and microglia. Exp Neurol.

[156] Robertson NJ, Faulkner S, Fleiss B, Bainbridge A, Andorka C, Price D, Powell E, Lecky-Thompson L, Thei L, Chandrasekaran M, Hristova M, Cady EB, Gressens P, Golay X, Raivich G (2013). Melatonin augments hypothermic neuroprotection in a perinatal asphyxia model. Brain.

[157] Wong C-S, Jow G-M, Kaizaki A, Fan L-W, Tien L-T (2014). Melatonin ameliorates brain injury induced by systemic lipopolysaccharide in neonatal rats. Neuroscience.

[158] Gressens P, Schwendimann L, Husson I, Sarkozy G, Mocaer E, Vamecq J, Spedding M (2008). Agomelatine, a melatonin receptor agonist with 5-HT(2C) receptor antagonist properties, protects the developing murine white matter against excitotoxicity. Eur J Pharmacol.

[159] Balduini W, Carloni S, Perrone S, Bertrando S, Tataranno ML, Negro S, Proietti F, Longini M, Buonocore G (2012). The use of melatonin in hypoxic-ischemic brain damage: an experimental study. J Matern Fetal Neonatal Med.

[160] Guven A, Uysal B, Gundogdu G, Oztas E, Ozturk H, Korkmaz A (2011). Melatonin ameliorates necrotizing enterocolitis in a neonatal rat model. J Pediatr Surg.

[161] Cekmez F, Cetinkaya M, Tayman C, Canpolat FE, Kafa IM, Uysal S, Tunc T, Sarıcı SÜ (2013). Evaluation of melatonin and prostaglandin E1 combination on necrotizing enterocolitis model in neonatal rats. Regul Pept.

[162] Xiong T, Qu Y, Mu D, Ferriero D (2011). Erythropoietin for neonatal brain injury: opportunity and challenge. Int J Dev Neurosci.

[163] Mohamad O, Chen D, Zhang L, Hofmann C, Wei L, Yu SP (2011). Erythropoietin reduces neuronal cell death and hyperalgesia induced by peripheral inflammatory pain in neonatal rats. Mol Pain.

[164] Liu W, Shen Y, Plane JM, Pleasure DE, Deng W (2011). Neuroprotective potential of erythropoietin and its derivative carbamylated erythropoietin in periventricular leukomalacia. Exp Neurol.

[165] Juul S (2012). Neuroprotective role of erythropoietin in neonates. J Matern Fetal Neonatal Med.

[166] Gitto E, Karbownik M, Reiter RJ, Tan DX, Cuzzocrea S, Chiurazzi P, Cordaro S, Corona G, Trimarchi G, Barberi I (2001). Effects of melatonin treatment in septic newborns. Pediatr Res.

[167] Merchant N. Melatonin as a novel neuroprotectant in preterm infants - trial study. ISRCTN registry. DOI 10.1186/isrctn15119574. http://www.isrctn.com/ISRCTN15119574 (2011). Accessed 17 Mar 2015.

[168] Merchant NM, Azzopardi DV, Counsell S, Gressens P, Dierl A, Gozar I et al. O-057 Melatonin As A Novel Neuroprotectant In Preterm Infants – A Double Blinded Randomised Controlled Trial (mint Study). Arch Dis Child. 2014;99:A43.

[169] Leuchter RH-V, Gui L, Poncet A, Hagmann C, Lodygensky GA, Martin E, Koller B, Darqué A, Bucher HU, Hüppi PS (2014). Association between early administration of high-dose erythropoietin in preterm infants and brain MRI abnormality at term-equivalent age. JAMA.

[170] O’Gorman RL, Bucher HU, Held U, Koller BM, Hüppi PS, Hagmann CF, Swiss EPO, Neuroprotection Trial Group (2015). Tract-based spatial statistics to assess the neuroprotective effect of early erythropoietin on white matter development in preterm infants. Brain.

[171] Barrington KJ (2001). The adverse neuro-developmental effects of postnatal steroids in the preterm infant: a systematic review of RCTs. BMC Pediatr.

[172] Shinwell ES, Karplus M, Reich D, Weintraub Z, Blazer S, Bader D, Yurman S, Dolfin T, Kogan A, Dollberg S, Arbel E, Goldberg M, Gur I, Naor N, Sirota L, Mogilner S, Zaritsky A, Barak M, Gottfried E (2000). Early postnatal dexamethasone treatment and increased incidence of cerebral palsy. Arch Dis Child Fetal Neonatal Ed.

[173] Murphy BP, Inder TE, Huppi PS, Warfield S, Zientara GP, Kikinis R, Jolesz FA, Volpe JJ (2001). Impaired cerebral cortical gray matter growth after treatment with dexamethasone for neonatal chronic lung disease. Pediatrics.

[174] Wilson-Costello D, Walsh MC, Langer JC, Guillet R, Laptook AR, Stoll BJ, Shankaran S, Finer NN, Van Meurs KP, Engle WA, Das A, Eunice Kennedy Shriver National Institute of Child Health and Human Development Neonatal Research Network (2009). Impact of postnatal corticosteroid use on neurodevelopment at 18 to 22 months’ adjusted age: effects of dose, timing, and risk of bronchopulmonary dysplasia in extremely low birth weight infants. Pediatrics.

[175] Lodygensky GA, Rademaker K, Zimine S, Gex-Fabry M, Lieftink AF, Lazeyras F, Groenendaal F, de Vries LS, Hüppi PS (2005). Structural and functional brain development after hydrocortisone treatment for neonatal chronic lung disease. Pediatrics.

[176] Benders MJNL, Groenendaal F, van Bel F, Ha Vinh R, Dubois J, Lazeyras F, Warfield SK, Hüppi PS, de Vries LS (2009). Brain development of the preterm neonate after neonatal hydrocortisone treatment for chronic lung disease. Pediatr Res.

[177] Watterberg KL, Shaffer ML, Mishefske MJ, Leach CL, Mammel MC, Couser RJ, Abbasi S, Cole CH, Aucott SW, Thilo EH, Rozycki HJ, Lacy CB (2007). Growth and neurodevelopmental outcomes after early low-dose hydrocortisone treatment in extremely low birth weight infants. Pediatrics.

[178] Baud O, Alberti C. The PREMILOC Randomized Controlled Trial: early low-dose hydrocortisone improves survival without bronchopulmonary dysplasia in extremely preterm infants. Pediatric Academic Societies Annual Meeting, Baltimore, E-PAS2015:2765.1. http://www.abstracts2view.com/pas/view.php?nu=PAS15L1_2765.1 (2015). Accessed 18 Sep 2015.

